# Contrasting Responses of Soil Physicochemical Properties and Bacterial Communities to Understory Intercropping in *Idesia polycarpa* Plantations

**DOI:** 10.3390/microorganisms14081782

**Published:** 2026-08-13

**Authors:** Wenwen Zhong, Zuwei Hu, Can Cao, Xiaolong Hao, Tong Niu, Yanan Ma, Yulan Bei, Jiajia Hou, Yingjian Niu, Yaohui Liu, Yanmei Wang, Li Dai, Qifei Cai, Xiaodong Geng, Juan Wang, Yongyu Ren, Fangming Liu, Zhen Liu, Shunyang Yao, Dongfeng Yan, Jing He, Tailin Zhong, Zhi Li

**Affiliations:** 1 College of Forestry, Henan Agricultural University, Zhengzhou 450046, China; 2 College of Urban Construction, Zhejiang Shuren University, Shaoxing 312028, China; 3 Henan Province Engineering Technology Research Center for *Idesia*, Zhengzhou 450046, China; 4 Key Laboratory for *Idesia polycarpa*, National Forestry and Grassland Administration, Zhengzhou 450046, China

**Keywords:** *Idesia polycarpa*, understory intercropping, Yellow River floodplain, soil bacterial community, soil nutrients, functional potential

## Abstract

Understory intercropping may improve soils, but its effects on bacterial communities in *Idesia polycarpa* plantations remain unclear. Five patterns were established in the Yellow River floodplain: pure *I. polycarpa* and *I. polycarpa* intercropped with *Astragalus membranaceus*, *Ipomoea batatas*, *Salvia rosmarinus*, or *Arachis hypogaea*. Soil properties and bacterial communities were assessed using 16S rRNA gene sequencing, LEfSe, FAPROTAX, and redundancy analysis. Intercropping significantly affected soil electrical conductivity, water content, organic matter, alkali-hydrolyzable nitrogen, available phosphorus, and available potassium, but not pH or bulk density. *Salvia rosmarinus* intercropping had 14.3 g kg^−1^ organic matter and 123 mg kg^−1^ available potassium; *Arachis hypogaea* had 14.5 g kg^−1^ organic matter and 81.3 mg kg^−1^ alkali-hydrolyzable nitrogen; and *Ipomoea batatas* had 11.9% water content and 13.6 mg kg^−1^ available phosphorus. Alpha diversity did not differ significantly. PCoA suggested differentiation, but PERMANOVA based on Bray–Curtis dissimilarity was nonsignificant (*r*^2^ = 0.329, *p* = 0.083); LEfSe identified treatment-associated biomarkers. Chemoheterotrophy and aerobic chemoheterotrophy accounted for 42.6–45.7% and 20.4–26.2%, respectively. Alkali-hydrolyzable nitrogen was associated with genus-level variation (*r*^2^ = 0.440, *p* = 0.031). Understory intercropping improved soil conditions and may influence bacterial composition and predicted functional potential.

## 1. Introduction

Soil microorganisms are essential components of forest ecosystems and participate in organic matter decomposition, nutrient mineralization, carbon and nitrogen cycling, soil aggregate formation, and plant nutrient acquisition [1,2]. Soil microbial diversity is also closely associated with terrestrial ecosystem multifunctionality [3]. The structure and functional potential of soil microbial communities are strongly influenced by environmental factors such as soil pH, nutrient availability, and water conditions [4,5]. In addition, vegetation can shape soil microbial communities through litter inputs, rhizodeposition, root exudation, nutrient uptake, and modification of the root-zone microenvironment [6]. Therefore, changes in plant composition and management practices may affect soil microorganisms both directly, through plant-derived resource inputs, and indirectly, through alterations in soil physicochemical conditions.

Understory vegetation management and intercropping are widely used to improve plantation soil conditions, ecological functions, and land-use efficiency. Intercropping may alter microbial diversity, taxonomic composition, the relative abundance of specific taxa, enzyme activities, and functional potential related to organic matter decomposition and nutrient cycling. Intercropping medicinal plants in *Camellia oleifera* plantations altered soil nutrients, enzyme activities, and bacterial community structure [7]. Walnut-based intercropping systems also modified soil bacterial communities [8], while grass intercropping in walnut orchards affected rhizosphere microbial composition and soil ecosystem functions [9]. In poplar plantations, intercropping altered microbial community composition without significantly affecting alpha diversity [10]. Studies of cereal–legume systems further showed that intercropping-induced changes in microbial communities were associated with improved nitrogen availability and assimilation [11], recruitment of beneficial bacteria and suppression of soil-borne pathogens [12], changes in root-exudate profiles [13], and enhanced microbiome functionality and soil nitrogen cycling [14]. These microbial responses may arise through both direct and indirect ecological mechanisms. Plant species differ in root exudates, rhizodeposition, and residue inputs, thereby altering the carbon substrates and signaling compounds available to rhizosphere microorganisms. Interspecific root interactions may further modify exudate profiles and selectively recruit beneficial microorganisms or suppress soil-borne pathogens [6,12,13]. Intercropping can also change soil moisture, aeration, pH, organic matter, and nutrient availability through complementary or competitive resource use and, in legume-based systems, biological nitrogen fixation. These changes may influence microbial community assembly and nutrient-cycling potential [11,14]. However, the magnitude and direction of microbial responses depend on plant identity, soil conditions, stand age, management duration, and environmental conditions. Consequently, intercropping may alter specific microbial taxa and functional potential before significant changes in overall microbial diversity become detectable [10].

Against this broader ecological background, *Idesia polycarpa* Maxim. is a woody oil and ecological–economic tree species that has received increasing attention in China. Its fruits and seeds are rich in oil, particularly unsaturated fatty acids, and previous studies have demonstrated that *I. polycarpa* fruit oil has a favorable fatty acid composition, abundant bioactive compounds, antioxidant capacity, and considerable development potential [15,16]. In addition, *I. polycarpa* grows rapidly, has strong environmental adaptability, and possesses a well-developed root system, giving it potential value in plantation establishment, ecological restoration, and woody oil production. Transcriptomic analysis has identified candidate genes involved in lipid biosynthesis in *I. polycarpa* fruits [17], while field investigations have shown that mature *I. polycarpa* plantations can maintain favorable growth and fruiting performance under suitable site conditions [18]. With the expansion of *I. polycarpa* cultivation, increasing attention has been paid to cultivation management, nutrient regulation, and belowground ecological processes. Li et al. [19] identified a core set of 727 bacterial OTUs shared among *I. polycarpa* rhizosphere samples, with Pseudomonadota, Acidobacteriota, and Actinobacteriota as the dominant phyla. At flowering, *Arthrobacter* and Micrococcaceae were enriched in female rhizospheres, whereas *Pedomicrobium* was the principal indicator taxon in male rhizospheres. Phosphorus and nitrogen fertilization have also been shown to affect seedling growth, soil physicochemical properties, and rhizosphere microbial communities [20,21]. Nevertheless, previous studies have mainly focused on fruit oil quality, mature plantation growth, fertilization, or sex-related differences, whereas soil bacterial communities and predicted functional potential under understory intercropping in young *I. polycarpa* plantations remain poorly understood.

The Yellow River floodplain is an ecologically sensitive region in northern China, where plantations contribute to wind protection, soil and water conservation, ecological restoration, and integrated land use. However, the predominantly sandy soils in parts of this region generally have limited water- and nutrient-retention capacities, which may constrain plantation establishment and soil ecological functioning. Previous studies have shown that land-use patterns and regional environmental conditions can substantially affect soil microbial biomass, community composition, and nutrient cycling in the Yellow River floodplain and irrigation areas [22,23]. Clear differences in soil nutrients, bacterial community composition, and predicted functions have also been detected among farmland, grassland, abandoned land, and bamboo forest soils in the Luo River Basin [24].

Nevertheless, little is known about how different understory plant configurations influence soil properties, bacterial communities, and predicted functional potential in young *I. polycarpa* plantations established in the Yellow River floodplain. We hypothesized that understory species representing contrasting functional types would create species-specific soil nutrient and water conditions and thereby induce corresponding changes in bacterial community composition, the enrichment of particular bacterial taxa, and predicted functional potential. To test this hypothesis, we compared a pure *I. polycarpa* plantation with four intercropping patterns involving *Astragalus membranaceus*, *Ipomoea batatas*, *Salvia rosmarinus*, and *Arachis hypogaea*. Soil physicochemical properties were measured to characterize changes in the soil environment, and 16S rRNA gene sequencing was used to assess bacterial diversity and community composition. LEfSe was used to identify treatment-associated bacterial taxa, FAPROTAX was used to characterize predicted functional potential, and redundancy analysis was conducted to explore associations between bacterial communities and soil environmental factors. The objectives were to determine the effects of different understory plant species on soil nutrient and water conditions; Evaluate changes in bacterial community composition and structure; Identify potential bacterial biomarkers associated with different planting patterns; Characterize predicted functional potential and its relationships with soil environmental factors. The findings may provide a theoretical basis for understory plant selection, soil quality improvement, and sustainable management of young *I. polycarpa* plantations in the Yellow River floodplain.

## 2. Materials and Methods

### 2.1. Study Site and Experimental Design

This study was conducted in an *I. polycarpa* plantation at Neihuang Forest Farm, Neihuang County, Anyang City, Henan Province, China (35°40′–35°56′ N, 114°35′–114°49′ E). The study area is located in the Yellow River floodplain at the eastern foot of the Taihang Mountains and is widely covered by Quaternary loess. The terrain is generally flat, with undulating sand dunes and scattered small dunes. The soil is mainly sandy, with relatively weak water- and nutrient-retention capacities and a certain degree of ecological vulnerability. The region has a warm-temperate, semi-humid continental monsoon climate, with an annual mean temperature of 13.6 °C. The mean temperature is −2.2 °C in January and 27.0 °C in July. The mean annual precipitation is approximately 600 mm, mainly concentrated from June to August. The experimental site was used to investigate understory compound planting patterns and their soil biological effects in young *I. polycarpa* plantations. During the experimental period, all treatments were established under the same site conditions and managed using consistent field management practices to minimize the influence of management differences on the experimental results.

Two-year-old *I. polycarpa* seedlings were used as the experimental tree material and were transplanted on 15 March 2025. The four understory species were selected to represent contrasting functional types: the legumes *Astragalus membranaceus* and *Arachis hypogaea*, the ground-cover crop *Ipomoea batatas*, and the aromatic herb *Salvia rosmarinus*. All have economic value and allow comparison of their effects on soil properties and bacterial communities in *I. polycarpa* plantations. A randomized block design was adopted, with five treatments: IP, pure *I. polycarpa* plantation; IP-AM, *I. polycarpa* + *Astragalus membranaceus* Fisch. ex Bunge; IP-IB, *I. polycarpa* + *Ipomoea batatas* (L.) Lam.; IP-SR, *I. polycarpa* + *Salvia rosmarinus* Spenn.; and IP-AH, *I. polycarpa* + *Arachis hypogaea* L. Each treatment had three replicates. The spacing of *I. polycarpa* was 3 m × 4 m, and each plot covered approximately 96 m^2^. Each plot contained 15 *I. polycarpa* seedlings arranged in three rows, with five seedlings per row. All plots were managed uniformly throughout the experimental period. Supplemental irrigation was applied according to soil moisture conditions, without additional irrigation for individual treatments. Weeds were removed manually, and no herbicides were used. No chemical pesticides were applied; pest and disease occurrence was monitored under natural field conditions.

### 2.2. Soil Sample Collection and Preservation

Soil samples were collected from *I. polycarpa* plantations under different understory planting treatments at the end of the growing season. Before sampling, surface litter, stones, and visible plant residues were carefully removed. The three replicates of each treatment corresponded to three spatially independent plots. Within each plot, five sampling points were distributed across the interior of the plot, while plot edges and visibly abnormal areas were avoided. At each point, an *I. polycarpa* tree with a similar growth status was selected, and one soil core was collected from the 0–20 cm soil layer within its canopy projection area using a sterilized soil auger. The five soil cores from each plot were combined in equal proportions and thoroughly homogenized to form one composite sample. Therefore, one independent composite sample was obtained from each plot, resulting in three biological replicates per treatment and a total sample size of 15 soil samples across the five treatments (*N* = 15). Each composite sample was divided into two subsamples: one was stored at −80 °C for microbial DNA extraction, and the other was air-dried for soil physicochemical analyses.

### 2.3. Determination of Soil Physicochemical Properties

Soil physicochemical properties were determined using standard soil agrochemical analysis methods [25]. Soil pH was measured using a pH meter (LC-PH-3S, Shanghai LichenBangxi Instrument Equipment Co., Ltd., Shanghai, China) after shaking a soil–water suspension at a ratio of 1:2.5 for 30 min. Soil electrical conductivity (EC) was measured using a conductivity meter (Shanghai INESA Scientific Instrument Co., Ltd., Shanghai, China) after extraction with deionized water. Soil water content (SWC) was determined by oven-drying fresh soil at 105 °C to constant weight. Soil bulk density (SBD) was measured using the cutting-ring method. Soil organic matter (SOM) was determined using the potassium dichromate oxidation–external heating method. Alkali-hydrolyzable nitrogen (AN) was measured using the alkaline hydrolysis diffusion method. Available phosphorus (AP) was extracted with NaHCO_3_ and determined using the molybdenum–antimony colorimetric method. Available potassium (AK) was extracted with NH_4_OAc and determined by flame photometry.

### 2.4. Soil DNA Extraction and PCR Amplification

Approximately 0.2–0.5 g of each frozen soil sample was promptly transferred into a centrifuge tube containing extraction lysis buffer. The samples were mechanically ground at 60 Hz using a Tissuelyser-48 multi-sample tissue grinder (Shanghai Jingxin, Shanghai, China) before total microbial DNA extraction. Total microbial DNA was extracted using the MagBeads FastDNA Kit for Soil (MP Biomedicals, Irvine, CA, USA) according to the manufacturer’s instructions. DNA integrity was assessed using 0.8% agarose gel electrophoresis, and DNA concentration and purity were determined using a NanoDrop NC2000 spectrophotometer (Thermo Fisher Scientific, Waltham, MA, USA). The V3–V4 hypervariable region of the bacterial 16S rRNA gene was amplified using the universal primers 338F and 806R. The primer sequences were 338F, 5′-ACTCCTACGGGAGGCAGCA-3′, and 806R, 5′-GGACTACHVGGGTWTCTAAT-3′ [26]. Sample-specific barcode sequences were incorporated into the primers to distinguish samples within the same sequencing library. The exact primer sequences used in the experiment are consistent with those reported for amplification of the bacterial 16S rRNA V3–V4 region. PCR amplification was performed using Q5 High-Fidelity DNA Polymerase. Each 25 μL reaction contained 1× Reaction Buffer, 1× High GC Buffer, 200 μM of each dNTP, 0.4 μM of each primer, 0.02 U μL^−1^ Q5 High-Fidelity DNA Polymerase (0.5 U per reaction), 2 μL of template DNA, and nuclease-free water to a final volume of 25 μL. The amplification conditions were as follows: initial denaturation at 98 °C for 5 min; 25 cycles of denaturation at 98 °C for 30 s, annealing at 52 °C for 30 s, and extension at 72 °C for 45 s; followed by a final extension at 72 °C for 5 min and holding at 12 °C. PCR products were examined using 2% agarose gel electrophoresis, and the target bands were excised and purified using an Axygen Gel Recovery Kit. Purified amplicons were quantified using the Quant-iT PicoGreen dsDNA Assay Kit and pooled in equimolar amounts. Sequencing libraries were constructed using the TruSeq Nano DNA LT Library Prep Kit (Illumina, San Diego, CA, USA). After library quality assessment, paired-end sequencing was performed on an Illumina NovaSeq 6000 platform (Illumina, San Diego, CA, USA) using the NovaSeq 6000 SP Reagent Kit, with a read length of 2 × 250 bp. DNA extraction, PCR amplification, library construction, and sequencing were conducted by Shanghai Personal Biotechnology Co., Ltd. (Shanghai, China).

### 2.5. Data Processing and Statistical Analysis

Raw paired-end reads were processed using QIIME 2 (2024.5) [27]. Raw reads were imported into a QIIME 2-compatible format and demultiplexed using the demux plugin. Primer sequences were removed using the q2-cutadapt trim-paired method [28], and reads without matching primer sequences were discarded. Quality filtering, denoising, paired-end read merging, and chimera removal were subsequently performed using the q2-dada2 denoise-paired method [29]. The trim-left-f and trim-left-r parameters were both set to 0, whereas the forward and reverse reads were truncated to 222 and 230 bp, respectively. The resulting ASVs and corresponding abundance table were generated, and the representative ASV sequences, approximately 405–430 bp in length, were taxonomically assigned against the SILVA Release 138 database [30]. Based on the ASV abundance table and taxonomic annotation results, bacterial community composition was analyzed and visualized at the phylum and genus levels. The normality of residuals and homogeneity of variances for soil physicochemical variables were evaluated using the Shapiro–Wilk and Levene’s tests, respectively. Variables satisfying these assumptions were analyzed using one-way ANOVA. Alpha diversity indices and predicted functional categories were compared using the Kruskal–Wallis test, with Benjamini–Hochberg false discovery rate correction applied to multiple comparisons of functional categories. Alpha diversity indices, including Chao1, Observed species, Shannon, Simpson, Pielou’s evenness, Faith’s phylogenetic diversity, and Good’s coverage, were calculated using QIIME 2 (2024.5) and R (4.3.3) software. Rarefaction curves were generated to evaluate sequencing depth. To reduce the influence of unequal sequencing depth among samples, the ASV abundance table was rarefied before alpha diversity analysis. Beta diversity was assessed using principal coordinate analysis based on the Bray–Curtis dissimilarity matrix [31]. Permutational multivariate analysis of variance (PERMANOVA) with 999 permutations was used to evaluate differences in bacterial community composition among understory planting treatments [32]. Linear discriminant analysis effect size (LEfSe) was used to identify differentially enriched bacterial taxa, with an LDA score threshold of 2.0 [33]. Based on the taxonomic annotation of ASVs, FAPROTAX was used to predict the potential ecological functions of soil bacterial communities [34,35]. Redundancy analysis was used to explore the relationships between bacterial community composition and soil physicochemical factors. Statistical analyses and figure generation were performed using IBM SPSS Statistics 26.0, Origin 2024, QIIME 2 (2024.5), and R (4.3.3). The ggplot2 package was used for visualization, and the ape package was used for principal coordinate analysis. Unless otherwise stated, statistical significance was determined at *p* < 0.05.

## 3. Results

### 3.1. Effects of Different Understory Intercropping Patterns on Soil Physicochemical Properties

Different understory planting treatments affected soil physicochemical properties to varying degrees in young *I. polycarpa* plantations (Table 1). One-way ANOVA showed that EC, SWC, SOM, AN, AP, and AK differed significantly among treatments (*p* < 0.05), whereas pH and SBD showed no significant differences (*p* > 0.05). These results indicate that understory compound planting mainly affected soil nutrient supply and water conditions, while its effects on soil acidity and bulk density were relatively limited. Among the treatments, IP-SR showed pronounced effects on soil EC, SOM, and AK. The EC and AK values in IP-SR reached 1.10 and 123 mg/kg, respectively, both of which were significantly higher than those in the other treatments. In addition, the SOM content in IP-SR was 14.3 g/kg, which was significantly higher than that in IP and IP-IB. IP-AH performed well in terms of SOM and AN, with values of 14.5 g/kg and 81.3 mg/kg, respectively. The AN content in IP-AH and IP-AM remained at relatively high levels and was significantly higher than that in IP. IP-IB showed higher SWC and AP values, reaching 11.9% and 13.6 mg/kg, respectively, suggesting that *Ipomoea batatas* understory planting may be more favorable for increasing soil water content and available phosphorus. In contrast, IP generally showed lower levels of SOM, AN, and AP. Overall, understory compound planting improved soil nutrient and water conditions in young *I. polycarpa* plantations, but the effects varied among understory plant species. IP-SR mainly increased EC, SOM, and AK; IP-AH mainly improved SOM and AN; IP-IB was more favorable for increasing SWC and AP; and IP-AM was mainly characterized by a higher AN level.

### 3.2. ASV Distribution Among Understory Intercropping Treatments

**Figure 1:**
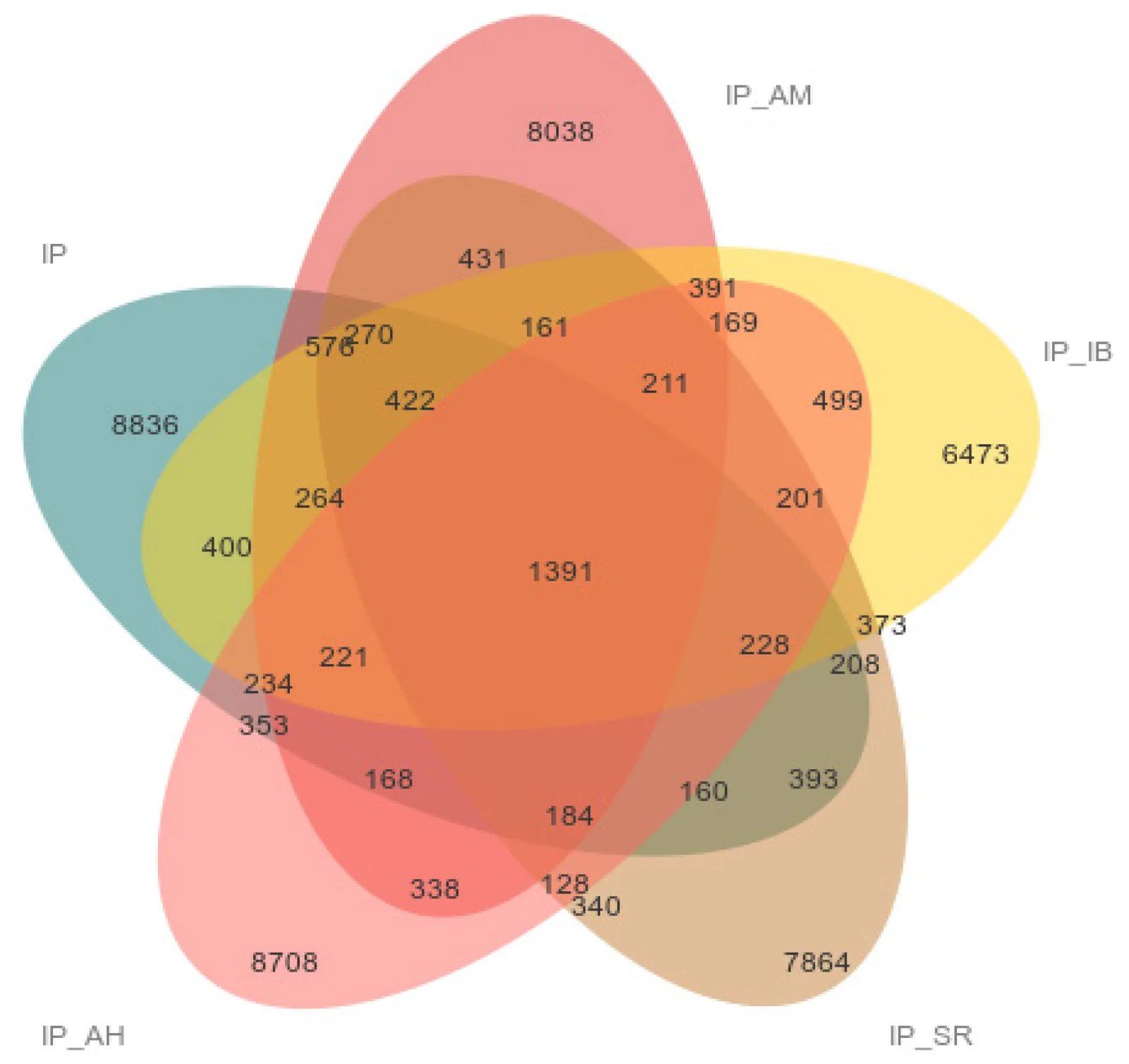
Venn diagram showing shared and treatment-specific amplicon sequence variants (ASVs) among the five understory intercropping treatments. IP, pure *I. polycarpa* plantation; IP-AM, *I. polycarpa* + *Astragalus membranaceus*; IP-IB, *I. polycarpa* + *Ipomoea batatas*; IP-SR, *I. polycarpa* + *Salvia rosmarinus*; IP-AH, *I. polycarpa* + *Arachis hypogaea*.

**Figure A1:**
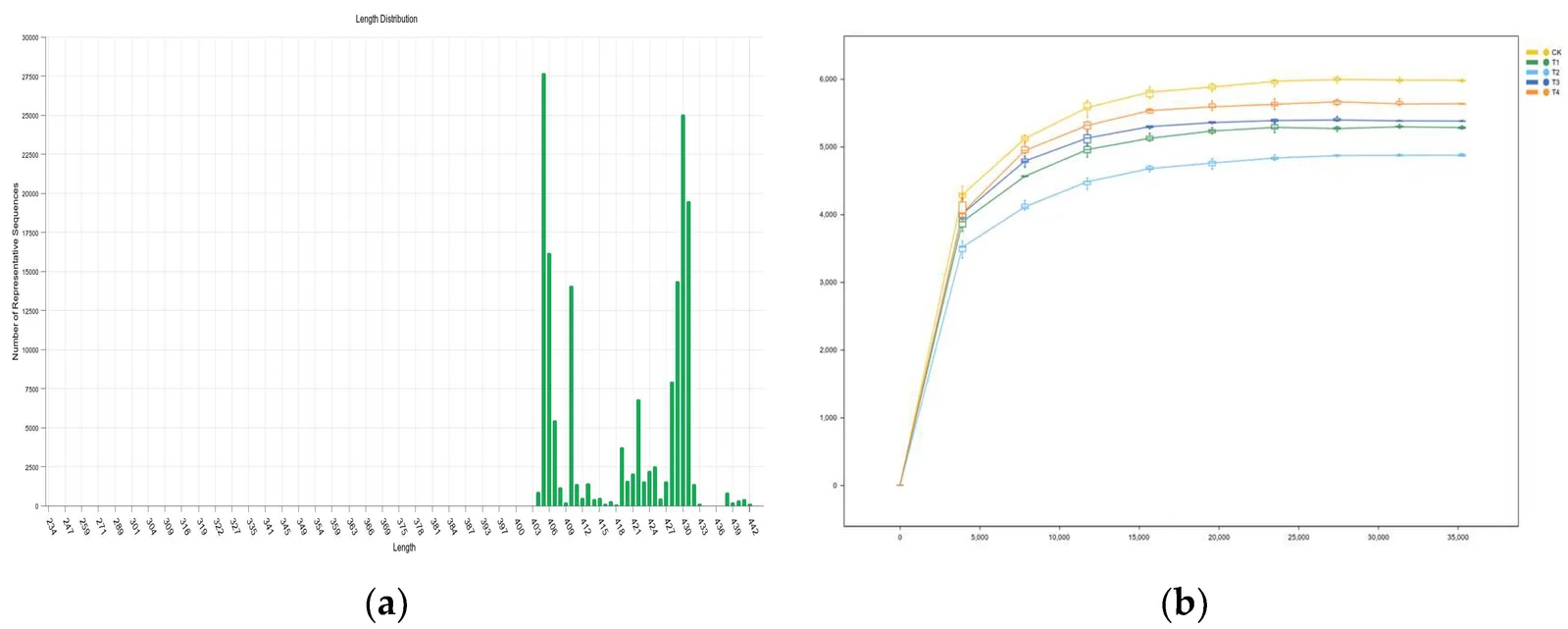
Sequencing characteristics of soil bacterial communities under different understory intercropping treatments. (**a**) Length distribution of representative sequences; (**b**) rarefaction curves of bacterial amplicon sequence variants (ASVs).

For the Venn analysis, ASV counts for each treatment were based on the pooled ASV data from three independently sequenced biological replicates. A total of 48,633 ASVs were detected across the five treatments. The numbers of ASVs detected in IP, IP-AM, IP-IB, IP-SR, and IP-AH were 14,308, 13,363, 11,846, 12,965, and 13,533, respectively. Among these, 1391 ASVs were shared by all five treatments, accounting for 2.86% of the total ASVs (Figure 1). The numbers of treatment-specific ASVs were 8836, 8038, 6473, 7864, and 8708 in IP, IP-AM, IP-IB, IP-SR, and IP-AH, respectively. The relatively low proportion of shared ASVs and the high numbers of treatment-specific ASVs indicate considerable variation in ASV occurrence among treatments. This pattern is consistent with the high spatial heterogeneity typically observed in sandy soils of the Yellow River floodplain. Detailed information on representative sequence-length distributions and taxonomic assignment statistics is provided in Figure A1a and Table A1, respectively.

### 3.3. Bacterial Alpha Diversity and Community Structure Variation

**Figure 2:**
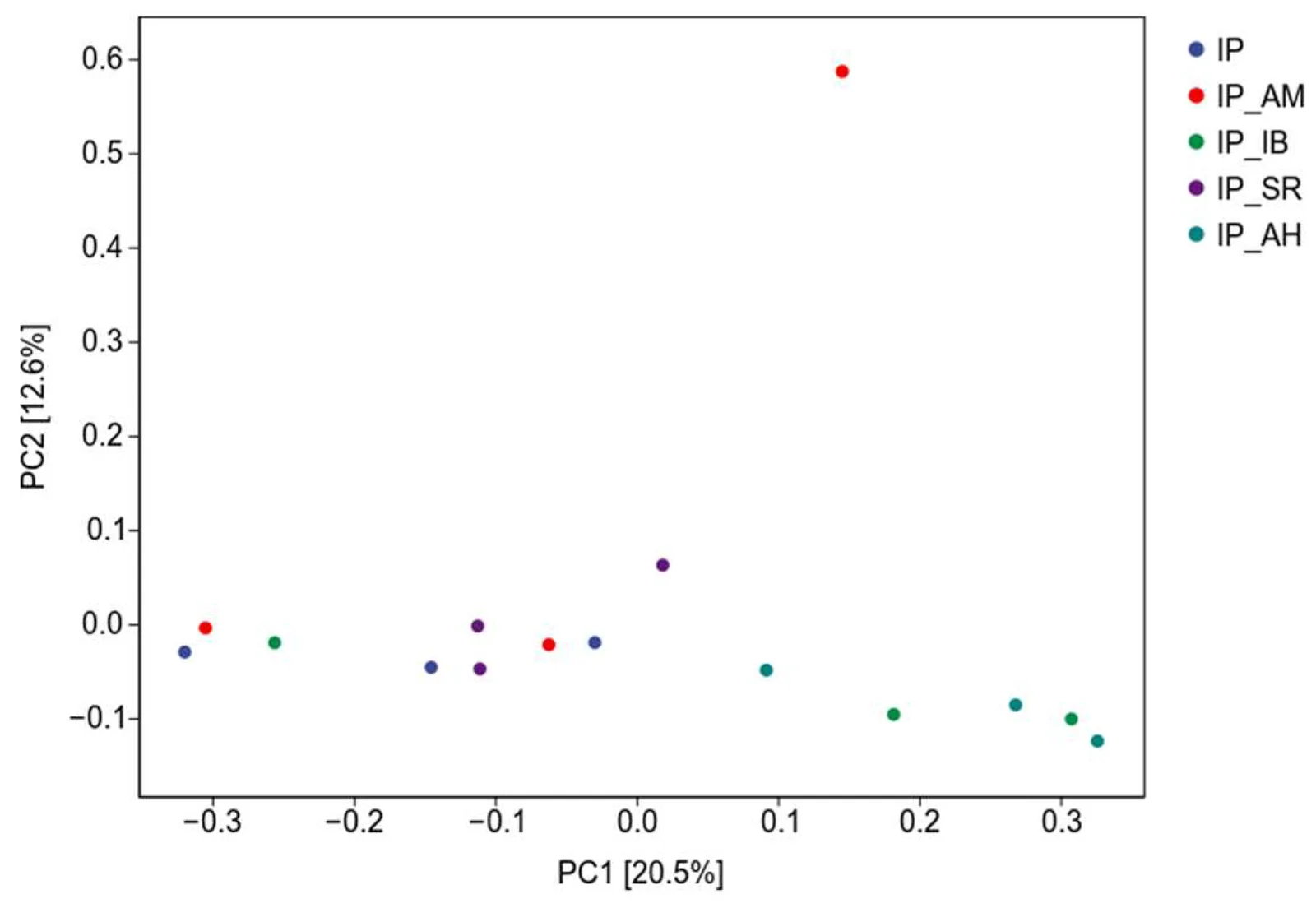
Principal coordinate analysis (PCoA) of soil bacterial communities under different understory planting treatments based on Bray–Curtis distance. IP, pure *I. polycarpa* plantation; IP-AM, *I. polycarpa* + *Astragalus membranaceus*; IP-IB, *I. polycarpa* + *Ipomoea batatas*; IP-SR, *I. polycarpa* + *Salvia rosmarinus*; IP-AH, *I. polycarpa* + *Arachis hypogaea*.

Rarefaction curves for all samples gradually approached a plateau (Figure A1b), indicating that the sequencing depth was sufficient for subsequent bacterial alpha diversity analyses. Soil bacterial alpha diversity indices showed no significant differences among different understory planting treatments. The Chao1, Observed species, Shannon, Simpson, Pielou’s evenness, and Faith’s PD indices did not differ significantly among treatments (*p* > 0.05), indicating that understory compound planting had limited effects on soil bacterial richness, evenness, and overall alpha diversity. PCoA based on Bray–Curtis distance showed a certain separation trend in soil bacterial community structure among treatments (Figure 2). PCoA1 and PCoA2 explained 20.5% and 12.6% of the total variation, respectively, with a cumulative explanation of 33.1%. In terms of sample distribution, IP samples were relatively clustered, whereas IP-IB and IP-AH samples were mostly distributed along the positive direction of PCoA1 and showed some distance from IP and IP-AM. IP-SR samples were mainly located in the central region of the ordination plot. PERMANOVA analysis showed that understory planting treatments explained 32.9% of the variation in bacterial community structure (*r*^2^ = 0.329), but the difference was not statistically significant (*p* = 0.083). Overall, understory compound planting did not significantly alter soil bacterial alpha diversity or overall community structure; however, the PCoA ordination indicated a certain differentiation trend among treatments, suggesting that understory plant configuration may have a potential regulatory effect on soil bacterial community structure.

### 3.4. Soil Bacterial Community Composition Under Different Understory Planting Treatments

**Figure 3:**
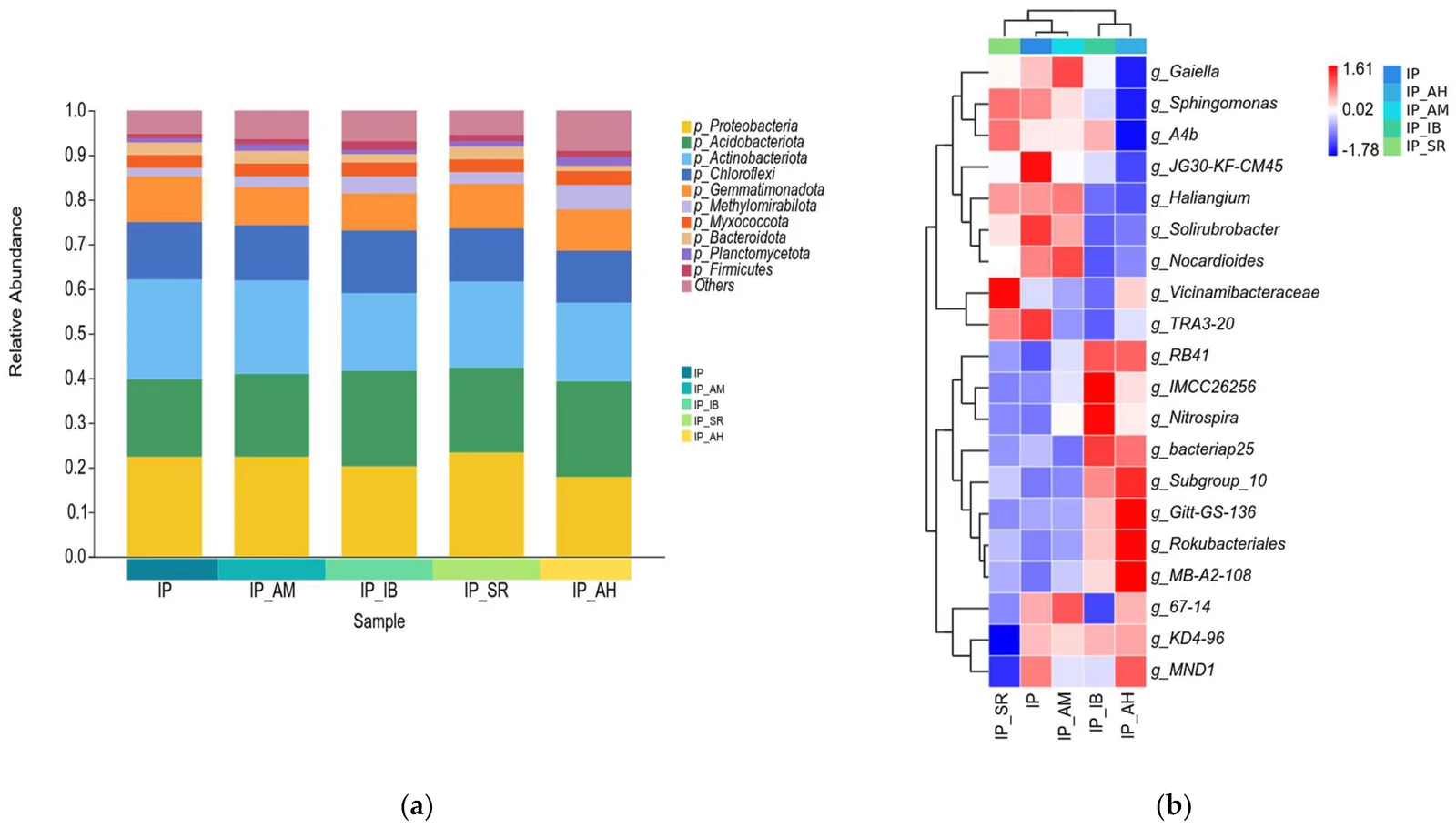
Soil bacterial community composition under different understory planting treatments. (**a**) Relative abundance of dominant bacterial phyla; (**b**) heatmap of the top 20 dominant bacterial genera. In (**b**), the color gradient represents standardized relative abundance, with red indicating higher relative abundance and blue indicating lower relative abundance. IP, pure *I. polycarpa* plantation; IP-AM, *I. polycarpa* + *Astragalus membranaceus*; IP-IB, *I. polycarpa* + *Ipomoea batatas*; IP-SR, *I. polycarpa* + *Salvia rosmarinus*; IP-AH, *I. polycarpa* + *Arachis hypogaea*.

Soil bacterial communities under different understory planting treatments showed similar dominant phylum-level compositions, but the relative abundances of the major phyla varied among treatments (Figure 3a). Proteobacteria, Acidobacteriota, Actinobacteriota, Chloroflexi, and Gemmatimonadota were the dominant phyla shared by all treatments. Among them, the relative abundance of Proteobacteria ranged from 17.7% to 23.3%, with the highest value observed in IP-SR and the lowest in IP-AH. Acidobacteriota accounted for 17.6–21.5% of the bacterial communities and was relatively more abundant in IP-IB and IP-AH. Actinobacteriota accounted for 17.5–22.3%, with relatively higher abundances in IP and IP-AM. Chloroflexi and Gemmatimonadota also accounted for certain proportions in all treatments, jointly constituting the main components of soil bacterial communities in *I. polycarpa* plantations. In terms of treatment-related differences, IP and IP-AM were mainly characterized by relatively higher abundances of Proteobacteria and Actinobacteriota, whereas IP-IB and IP-AH showed relatively higher abundances of Acidobacteriota. IP-SR was characterized by relatively higher abundances of Proteobacteria and Gemmatimonadota. In addition, the relative abundance of Methylomirabilota in IP-AH was higher than that in the other treatments. Overall, understory planting did not change the basic composition of dominant bacterial phyla, but it affected the relative abundances of some dominant phyla.

At the genus-annotation level, the top 20 dominant taxa collectively accounted for 34.9–40.4% of the bacterial communities across treatments, as shown in Figure 3b. RB41 was the most abundant taxon, with its mean relative abundance ranging from 4.60% in IP to 7.10% in IP-IB. Other dominant taxa included Vicinamibacteraceae, with relative abundances ranging from 4.10% to 5.00%; KD4-96, from 2.60% to 2.90%; Rokubacteriales, from 1.60% to 4.80%; MB-A2-108, from 2.20% to 3.20%; JG30-KF-CM45, from 1.70% to 2.90%; and *Sphingomonas*, from 0.60% to 2.40%. Differences in the distribution patterns of dominant taxa were also observed among treatments. RB41, IMCC26256, and *Nitrospira* showed their highest mean relative abundances in IP-IB, reaching 7.10%, 1.70%, and 1.20%, respectively. Rokubacteriales, MB-A2-108, Gitt-GS-136, and Subgroup_10 showed their highest mean relative abundances in IP-AH, reaching 4.80%, 3.20%, 2.30%, and 2.30%, respectively. Vicinamibacteraceae, *Sphingomonas*, and A4b showed their highest mean relative abundances in IP-SR, reaching 5.00%, 2.40%, and 1.30%, respectively. JG30-KF-CM45 was most abundant in IP, with a mean relative abundance of 2.90%. *Solirubrobacter* showed mean relative abundances of 1.40% in IP and 1.30% in IP-AM, whereas *Nocardioides* accounted for 1.30% and 1.40% in these two treatments, respectively. Overall, although the dominant bacterial composition at the phylum level was broadly similar among treatments, the relative abundance patterns of dominant taxa at the genus-annotation level varied among understory intercropping treatments.

### 3.5. Differential Taxa Identified by LEfSe Analysis

Taxonomic annotation was performed for the 1391 ASVs shared among all five treatments and for the ASVs specific to each treatment. Among the shared ASVs, 25.1% were assigned to Acidobacteriota, 21.5% to Proteobacteria, 18.5% to Actinobacteriota, 12.6% to Chloroflexi, and 10.7% to Gemmatimonadota. At the genus-annotation level, the most frequent assignments among the shared ASVs were RB41, Unclassified_f_Gemmatimonadaceae, and Vicinamibacteraceae, accounting for 8.48%, 7.30%, and 7.30%, respectively. The treatment-specific ASVs were also mainly assigned to Acidobacteriota and Proteobacteria, which accounted for 20.1–22.1% and 17.5–21.4% of the treatment-specific ASVs, respectively. At the genus-annotation level, Vicinamibacteraceae was the most frequent assignment among the ASVs specific to IP, IP-AM, and IP-SR, accounting for 5.70%, 5.50%, and 6.00%, respectively. Unclassified_o_Vicinamibacterales was the most frequent assignment among the IP-IB-specific ASVs, accounting for 5.70%, whereas Unclassified_f_Gemmatimonadaceae was the most frequent assignment among the IP-AH-specific ASVs, accounting for 6.30%.

**Figure 4:**
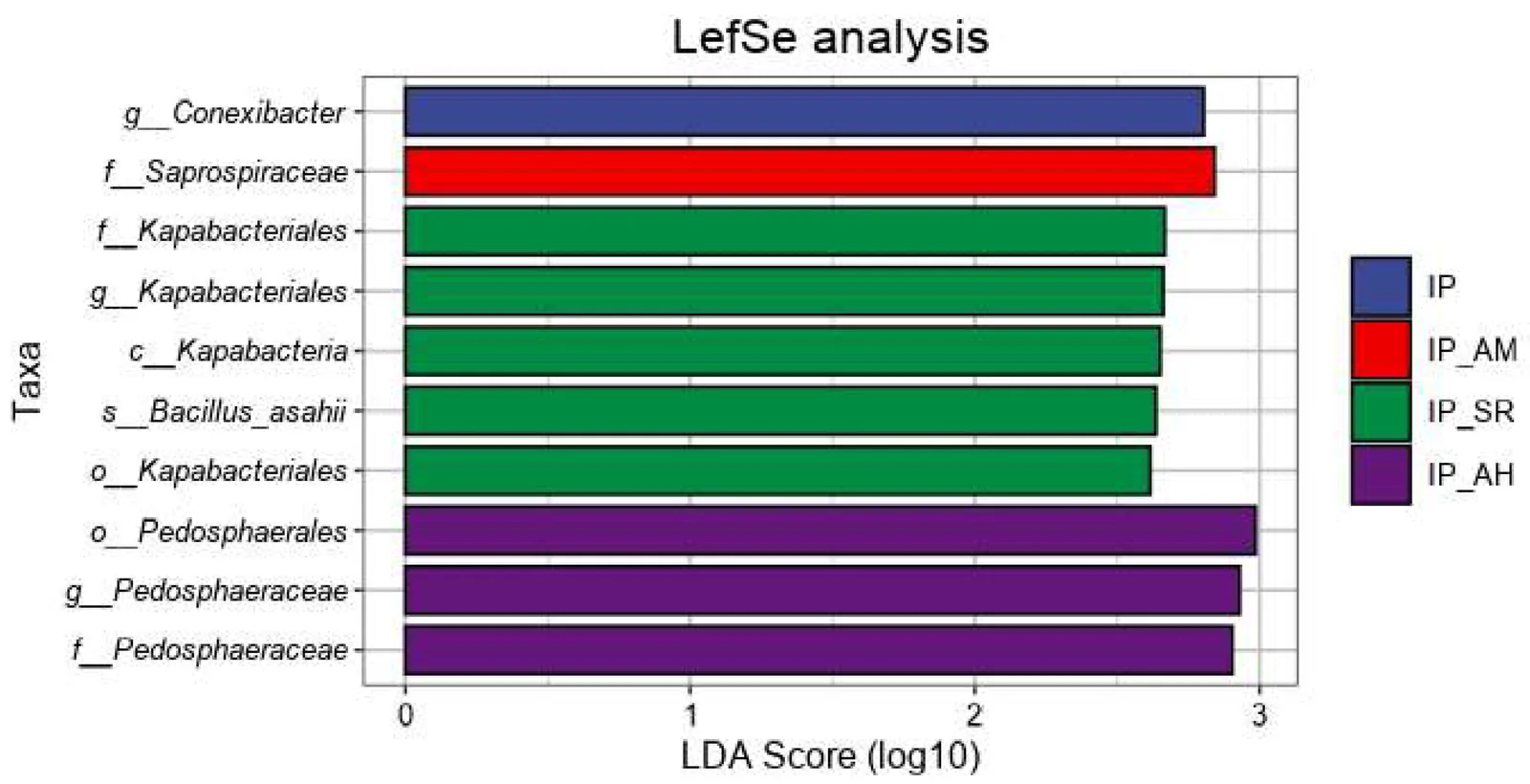
LEfSe analysis of differential bacterial taxa among different understory planting treatments. The histogram shows taxa with an LDA score greater than 2.0. Different colors indicate taxa enriched in different treatments. IP, pure *I. polycarpa* plantation; IP-AM, *I. polycarpa* + *Astragalus membranaceus*; IP-IB, *I. polycarpa* + *Ipomoea batatas*; IP-SR, *I. polycarpa* + *Salvia rosmarinus*; IP-AH, *I. polycarpa* + *Arachis hypogaea*.

With an LDA score threshold of 2.0, differentially enriched taxa were detected in IP, IP-AM, IP-SR, and IP-AH, whereas no biomarker taxa reaching the threshold were identified in IP-IB (Figure 4). This result may reflect a relatively mild selective effect of *Ipomoea batatas* intercropping on soil bacterial taxa during the first growing season. *Conexibacter* was identified as a biomarker of IP, with an LDA score of 2.9, while Saprospiraceae was associated with IP-AM, with an LDA score of 2.9. Kapabacteria, Kapabacteriales, and *Bacillus asahii* were biomarkers of IP-SR, with LDA scores ranging from 2.7 to 2.7. Pedosphaerales and Pedosphaeraceae were biomarkers of IP-AH, with LDA scores ranging from 2.9 to 3.0. Comparison of the Venn and LEfSe results showed that differential enrichment did not necessarily indicate treatment-specific occurrence. Among the treatment-specific ASVs, 18 IP-specific ASVs were assigned to *Conexibacter*, 15 IP-AM-specific ASVs to Saprospiraceae, 20 IP-SR-specific ASVs to Kapabacteria and Kapabacteriales, and 75 IP-AH-specific ASVs to Pedosphaerales and Pedosphaeraceae. However, these taxa were also detected in other treatments. The four ASVs assigned to *Bacillus asahii* included one ASV shared among all treatments and three ASVs detected in multiple, but not all, treatments; none was specific to IP-SR. Thus, treatment-specific ASVs identified by the Venn analysis represent differences in taxon occurrence, whereas LEfSe biomarkers represent differences in relative abundance among treatments.

### 3.6. Predicted Functional Potential of Soil Bacterial Communities

**Figure 5:**
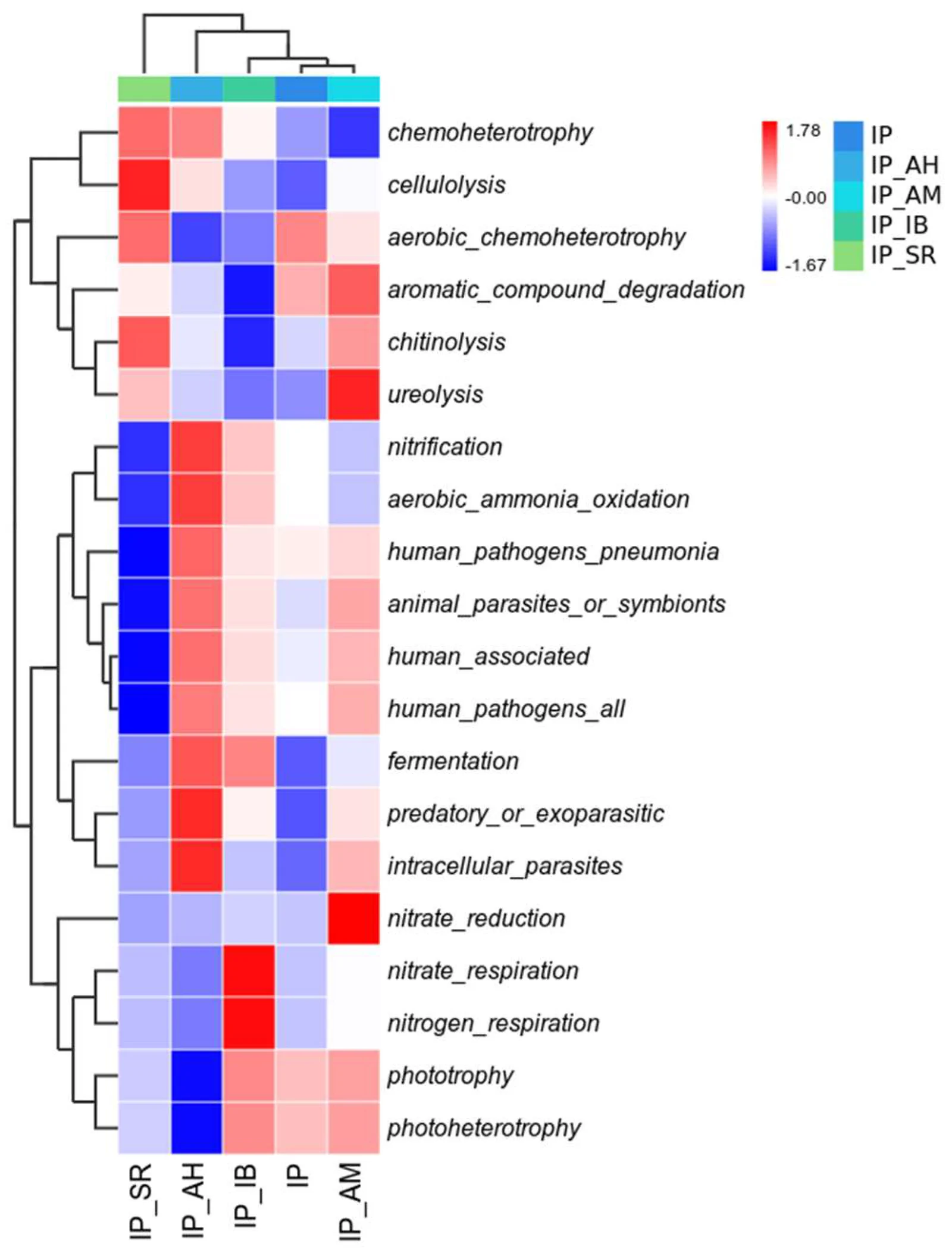
FAPROTAX-based heatmap of predicted functional profiles of soil bacterial communities under different understory planting treatments in young *I. polycarpa* plantations. Rows represent predicted functional groups, and columns represent different treatments. The color gradient indicates standardized relative abundance, with red representing higher relative abundance and blue representing lower relative abundance. IP, pure *I. polycarpa* plantation; IP-AM, *I. polycarpa* + *Astragalus membranaceus*; IP-IB, *I. polycarpa* + *Ipomoea batatas*; IP-SR, *I. polycarpa* + *Salvia rosmarinus*; IP-AH, *I. polycarpa* + *Arachis hypogaea*.

FAPROTAX was used to predict the potential ecological functions of soil bacterial communities under different understory intercropping treatments (Figure 5). The predicted functions were mainly related to carbon metabolism and nitrogen cycling. Chemoheterotrophy was the most abundant predicted functional category, accounting for 42.6–45.7% across treatments, followed by aerobic chemoheterotrophy, which accounted for 20.4–26.2%. These results indicated that chemoheterotrophic organic carbon utilization represented the dominant predicted functional potential of the soil bacterial communities. Numerical variation in several predicted functional categories was observed among treatments. Nitrification and aerobic ammonia oxidation showed their highest mean relative abundances in IP-AH and their lowest values in IP-SR. Nitrate reduction and ureolysis showed relatively high mean values in IP-AM, whereas nitrate respiration and nitrogen respiration were highest in IP-IB. Chemoheterotrophy, aerobic chemoheterotrophy, chitinolysis, and cellulolysis showed relatively high mean values in IP-SR. However, the overall predicted functional profiles did not differ significantly among treatments based on PERMANOVA using Bray–Curtis dissimilarities, with a *p* value of 0.090. Similarly, none of the 20 dominant predicted functional categories differed significantly among treatments according to Kruskal–Wallis tests after Benjamini–Hochberg false discovery rate correction, with all adjusted *p* values ≥ 0.05.

### 3.7. Relationships Between Soil Environmental Factors and Bacterial Communities

**Figure 6:**
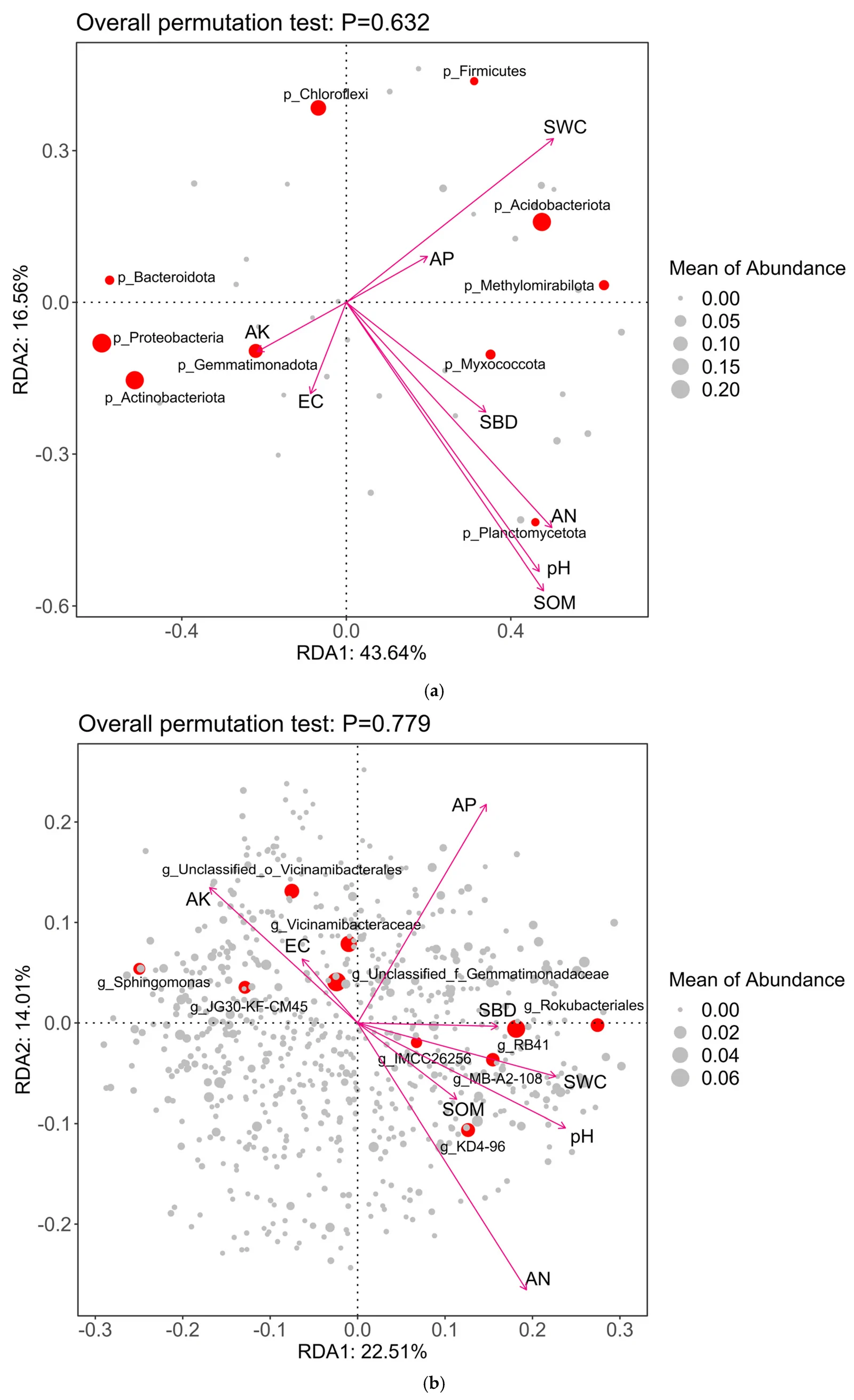
Redundancy analysis (RDA) showing the associations between soil environmental factors and bacterial community composition. (**a**) Phylum-level bacterial community composition; (**b**) genus-level bacterial community composition. Pink arrows represent soil environmental variables, and their direction and length indicate the direction and relative strength of the fitted associations. Red circles represent the major bacterial taxa, gray circles represent the remaining taxa, and circle size indicates mean relative abundance. The *p* values of the overall permutation tests are shown above each panel.

RDA was used to assess the relationships between soil environmental factors and bacterial community composition (Figure 6). At the phylum level, RDA1 and RDA2 explained 43.6% and 16.6% of the variation, respectively, accounting for 60.2% in total (Figure 6a). Although the overall permutation test was not significant (*p* = 0.632), SOM (*r*^2^ = 0.542, *p* = 0.009), pH (*r*^2^ = 0.516, *p* = 0.012), AN (*r*^2^ = 0.487, *p* = 0.020), and SWC (*r*^2^ = 0.436, *p* = 0.040) were significantly associated with phylum-level community distribution. SWC and AP were mainly associated with Acidobacteriota and Firmicutes, whereas SOM, pH, and AN were more closely aligned with Planctomycetota and Myxococcota. Proteobacteria, Actinobacteriota, Gemmatimonadota, and Bacteroidota were distributed mainly along the negative direction of RDA1, indicating different responses to the measured soil factors. At the genus level, RDA1 and RDA2 explained 22.5% and 14.0% of the variation, respectively, with a cumulative explanation of 36.5% (Figure 6b). The overall permutation test was also not significant (*p* = 0.779). AN was significantly associated with genus-level community distribution (*r*^2^ = 0.440, *p* = 0.031), while AP, pH, and SWC showed marginal associations. RB41, Rokubacteriales, MB-A2-108, KD4-96, MND1, and IMCC26256 were mainly associated with AN, SOM, SWC, pH, and SBD, whereas *Sphingomonas*, JG30-KF-CM45, and Vicinamibacteraceae were more closely related to AK and EC. Overall, phylum-level bacterial communities were mainly associated with SOM, pH, AN, and SWC, whereas genus-level variation was primarily related to AN. However, because the overall RDA models were not statistically significant at either the phylum level (*p* = 0.632) or the genus level (*p* = 0.779), these single-factor relationships should be regarded as exploratory associations rather than confirmed drivers of bacterial community structure.

## 4. Discussion

### 4.1. Effects of Understory Planting on Soil Physicochemical Properties

Understory intercropping significantly affected soil EC, SWC, SOM, AN, AP, and AK in young *I. polycarpa* plantations, whereas pH and SBD remained unchanged. This indicates that understory plants primarily influenced relatively dynamic soil properties, particularly nutrient availability and water conditions, while soil acidity and physical structure were less responsive during the experimental period. Differences in root distribution, nutrient acquisition, residue return, and rhizodeposition among plant species can alter resource availability and nutrient turnover in the root zone [36,37]. The effects varied among planting patterns. The *I. polycarpa*–*Salvia rosmarinus* pattern had relatively high SOM, AP, and AK, whereas the *I. polycarpa*–*Arachis hypogaea* pattern had high SOM and AN. The *I. polycarpa*–*Astragalus membranaceus* pattern was also characterized by relatively high AN. Both legume treatments had relatively high AN contents compared with the pure *I. polycarpa* plantation, although the difference between *Arachis hypogaea* and *Astragalus membranaceus* was not statistically significant. Their similar AN levels may reflect associations with nitrogen-fixing microorganisms, whereas the slight variation may be related to differences in rhizobial compatibility, nodulation, root traits, plant nitrogen demand, and rhizodeposit inputs. However, because nodulation and nitrogen-fixation rates were not measured, the observed AN patterns cannot be attributed solely to biological nitrogen fixation. Species-specific root metabolites may also influence the rhizosphere environment. In the related medicinal legume *Astragalus mongholicus*, changes in root-exudate composition were accompanied by shifts in root-associated microbial communities, and several root-derived metabolites showed inhibitory effects on the host plant, *Fusarium oxysporum*, or both [38]. This suggests that chemically specific root inputs may contribute to microbial responses in *Astragalus* systems. However, whether similar compounds affected bacterial communities under *Astragalus membranaceus* intercropping remains to be tested.

The *I. polycarpa*–*Ipomoea batatas* pattern showed higher SWC and AP, which may be associated with its ground-cover characteristics and root distribution. Compared with the pure *I*. *polycarpa* plantation, soil AK was significantly lower under intercropping with *Astragalus membranaceus*, *Ipomoea batatas*, and *Arachis hypogaea*, but significantly higher under intercropping with *Salvia rosmarinus*. The lower AK levels may reflect greater potassium uptake by the understory crops during the first growing season, together with treatment-specific differences in root-exudate chemistry and the limited return of potassium through litter at this early establishment stage. In contrast, the higher AK under *Salvia rosmarinus* intercropping may indicate a different balance among potassium uptake, mobilization, and return. The nonsignificant responses of pH and SBD may be related to the relatively short experimental duration, as more stable soil properties generally respond to sustained differences in organic inputs and management practices [39,40].

### 4.2. Responses of Soil Bacterial Diversity and Community Composition to Understory Planting

Bacterial alpha diversity did not differ significantly among planting patterns, and PERMANOVA detected no significant overall difference in community structure, although PCoA indicated a certain differentiation trend. In contrast, significant changes in microbial diversity and community composition have been reported in proso millet–mung bean intercropping and medicinal plant intercropping in *Camellia oleifera* plantations [7,11]. These contrasting results indicate that microbial responses to intercropping are context dependent and may vary with plant combinations, soil conditions, root interactions, and treatment duration. Significant shifts in soil microbiota are more likely when the associated crops differ markedly in root architecture, nutrient-acquisition strategies, and exudate composition, and when their rhizospheres overlap sufficiently to generate strong belowground interactions. Such effects may be further enhanced when intercropping substantially alters soil nutrient availability, moisture, pH, or organic carbon inputs, or when the planting duration is long enough for these changes to accumulate [13,32,33]. In the present study, the young stand age, uniform management, and short experimental period may have limited the development of significant community-level differences. The apparent stability of the bacterial community does not necessarily indicate functional insensitivity. Microbial resistance, resilience, and functional redundancy may maintain overall diversity and major ecological processes even when the relative abundances of individual taxa change [41,42]. Proteobacteria, Acidobacteriota, Actinobacteriota, Chloroflexi, and Gemmatimonadota remained dominant across all planting patterns, suggesting that the basic community framework was maintained by the shared sandy soil, weak alkalinity, regional climate, and field management [43,44]. Nevertheless, the relative abundances of several dominant groups varied among treatments. Proteobacteria and Gemmatimonadota were relatively more abundant under *I. polycarpa*–*Salvia rosmarinus* planting, Acidobacteriota under *I. polycarpa*–*Ipomoea batatas* and *I. polycarpa*–*Arachis hypogaea* planting, and Actinobacteriota in the pure *I. polycarpa* plantation and the *I. polycarpa*–*Astragalus membranaceus* pattern. Because Acidobacteriota includes diverse ecological strategies, its response cannot be explained solely by a simple copiotrophic–oligotrophic classification [45]. Forest soil bacterial communities often maintain stable dominant lineages while showing changes in their relative abundance in response to plant identity and resource inputs [46]. Thus, understory intercropping may have induced taxonomic and predicted functional adjustment within a broadly conserved community framework rather than causing a fundamental restructuring of the bacterial community. Root exudates may contribute to both the maintenance of shared microbiota and the recruitment of treatment-specific taxa. Because *I. polycarpa* was present in all treatments, common host-derived rhizodeposition and shared soil conditions may have favored bacterial taxa adapted to similar carbon substrates and root-zone environments. In contrast, species-specific exudates from the understory plants may have selectively recruited particular bacterial groups by providing distinct nutrients and chemical signals [13]. This mechanism may partly explain the coexistence of a shared bacterial community with treatment-associated differences in relative abundance and biomarkers. However, because root exudates were not directly measured, their contribution remains a hypothesis requiring metabolomic and microbial validation. The observed compositional trends may strengthen and become statistically significant as intercropping effects accumulate over successive growing seasons.

### 4.3. Differential Taxa and Their Ecological Implications

LEfSe analysis identified *Conexibacter* in the pure *I. polycarpa* plantation, Saprospiraceae under *I. polycarpa*–Astragalus membranaceus planting, Kapabacteria-related taxa under *I. polycarpa*–*Salvia rosmarinus* planting, and Pedosphaeraceae-related taxa under *I. polycarpa*–*Arachis hypogaea* planting. Thus, specific bacterial lineages responded to understory plant identity despite the absence of a significant overall community shift. *Conexibacter* belongs to Actinobacteriota, some members of which participate in the decomposition and transformation of plant-derived organic materials, particularly in less fertile soils [47]. Its enrichment in the pure *I. polycarpa* plantation may therefore be associated with the relatively low SOM, AN, and AP levels in this treatment. However, its specific ecological function cannot be inferred solely from higher-level taxonomy. Some members of Saprospiraceae can degrade complex organic substrates, and their enrichment under *I. polycarpa*–*Astragalus membranaceus* planting may be related to differences in root-derived carbon or residue composition [48]. Pedosphaeraceae belongs to Verrucomicrobiota, an abundant but historically under-recognized soil bacterial group [49]. Related lineages can utilize plant-derived polysaccharides such as xylan, suggesting a possible association with differences in organic substrate availability [50]. Although the IP-AH treatment had relatively high AN, neither *Rhizobium* nor *Bradyrhizobium* was identified as an enriched biomarker. This may be related to the sampling design, because composite near-root soil was collected within the canopy projection of *I. polycarpa*, rather than peanut nodules or soil tightly adhering to *Arachis hypogaea* roots. Rhizobia, particularly *Bradyrhizobium*, may be strongly concentrated within field-grown peanut nodules [51]. They may therefore have been diluted in the composite soil samples or failed to exceed the LEfSe threshold. Because nodulation and nitrogen-fixation activity were not measured, the absence of rhizobial biomarkers does not indicate the absence of peanut–*rhizobium* symbiosis. The greater number of Kapabacteria-related biomarkers under *I. polycarpa*–*Salvia rosmarinus* planting suggests a stronger selective effect of *Salvia rosmarinus* on particular bacterial groups. This response may partly reflect species-specific secondary metabolites. Rosemary is rich in terpenoid and phenolic compounds, and multiple terpenes have been detected in soils beneath rosemary plants [52]. These compounds may selectively influence rhizosphere microorganisms, although they may enter the soil through root exudation, litter leaching, or volatilization. Because root exudates and allelopathic activity were not measured in this study, this mechanism remains hypothetical. Moreover, Kapabacteria-related taxa should be regarded as potential planting-pattern biomarkers rather than assigned specific ecological functions.

### 4.4. Changes in the Predicted Functional Potential of Soil Bacterial Communities

FAPROTAX prediction showed that chemoheterotrophy and aerobic chemoheterotrophy were the dominant functional groups across planting patterns, indicating that organic carbon utilization was the principal predicted functional potential of the bacterial communities. Nitrogen-cycling functions also varied among treatments. Nitrification and aerobic ammonia oxidation were relatively higher under *I. polycarpa*–*Arachis hypogaea* planting, corresponding to its relatively high AN content. Ammonia oxidation is the first and often rate-limiting step of nitrification, but it is regulated by the abundance, physiology, and niche differentiation of ammonia-oxidizing bacteria and archaea [53]. Soil nitrogen cycling also comprises multiple interconnected microbial processes rather than isolated functional categories [54], and ammonia-oxidizing archaea may dominate ammonia oxidation in some soils [55]. Therefore, higher predicted nitrification potential cannot be directly interpreted as evidence of increased nitrification rates. Chemoheterotrophy, aerobic chemoheterotrophy, chitinolysis, and cellulolysis were relatively higher under *I. polycarpa*–*Salvia rosmarinus* planting, broadly corresponding to its higher SOM. This suggests a potentially greater capacity for carbon-substrate utilization and organic matter decomposition. However, taxonomic composition does not necessarily predict process rates because microbial functions are also influenced by environmental conditions, functional redundancy, and microbial activity [56]. Accordingly, FAPROTAX results should be interpreted as predicted functional potential rather than direct measurements of carbon decomposition or nitrogen transformation [34,35]. Future studies should validate these predictions by quantifying key functional genes, such as *amoA* for ammonia oxidation and *nifH* for biological nitrogen fixation, together with extracellular enzyme assays and direct measurements of nitrogen transformation rates.

### 4.5. Ecological Implications of Soil Environmental Factors for Bacterial Communities

Single-factor fitting showed that SOM, pH, AN, and SWC were significantly associated with phylum-level community distribution, whereas AN was significantly associated with genus-level variation. However, the overall RDA models were not significant. These fitted relationships should therefore be considered exploratory associations rather than evidence that the measured environmental variables independently determined bacterial community structure. Soil pH is an important correlate of bacterial biogeography because it affects nutrient solubility, microbial physiology, and habitat suitability [57,58]. Although pH did not differ significantly among planting patterns, small variations within the weakly alkaline range may still have been associated with the distribution of certain bacterial groups. This does not indicate that understory planting altered pH, but rather that existing pH variation was aligned with part of the community pattern. SOM provides carbon and energy for heterotrophic microorganisms, whereas AN represents the availability of mineralizable nitrogen. Nutrient enrichment can alter bacterial taxonomic and functional composition, although the response depends on ecosystem background and treatment duration [59,60]. The significant genus-level association with AN suggests that nitrogen availability may be particularly relevant to the distribution of some dominant taxa, but the RDA results cannot establish causality. Plant species differ in root-exudate composition and substrate supply, which can select microbial taxa according to their metabolic preferences [61]. Soil water availability may further regulate substrate diffusion, oxygen conditions, and microbial activity. Land use can alter the resistance and resilience of soil food webs to drought [62], while water limitation can delay root microbiome development and selectively enrich particular bacterial groups [63]. The association between SWC and phylum-level composition is therefore ecologically plausible, especially in the sandy soils of the Yellow River floodplain. Overall, understory intercropping produced clearer changes in soil nutrient and water conditions than in bacterial alpha diversity or overall community structure. Microbial responses were mainly reflected in changes in the relative abundance of dominant taxa, treatment-associated biomarkers, and predicted functional profiles. The *I. polycarpa*–*Salvia rosmarinus* pattern performed well in improving SOM, AP, and AK; the *I. polycarpa*–*Arachis hypogaea* pattern had relatively high SOM and AN; the *I. polycarpa*–*Ipomoea batatas* pattern improved SWC and AP; and the *I. polycarpa*–*Astragalus membranaceus* pattern maintained relatively high AN. Because the study covered only one developmental stage and included limited replication, future work should incorporate seasonal and interannual monitoring, greater spatial replication, soil enzyme activities, functional genes, and metagenomic analyses to determine whether these early responses lead to sustained improvements in soil quality and plantation performance.

## 5. Conclusions

This study showed that understory compound planting significantly altered soil nutrient and water conditions in *I. polycarpa* plantations in the Yellow River floodplain. Significant differences were observed in EC, SWC, SOM, AN, AP, and AK among planting patterns, whereas soil pH and bulk density remained relatively stable. The effects varied among understory plant species: *I. polycarpa*–*Salvia rosmarinus* planting performed well in increasing SOM, AP, and AK; *I. polycarpa*–*Arachis hypogaea* planting showed relatively high SOM and AN; *I. polycarpa*–*Ipomoea batatas* planting favored increases in SWC and AP; and *I. polycarpa*–*Astragalus membranaceus* planting was characterized by a relatively high AN level. Understory planting did not significantly affect soil bacterial alpha diversity, and the overall difference in community structure was also not statistically significant. However, the PCoA results indicated a certain trend of community differentiation among planting patterns. Proteobacteria, Acidobacteriota, Actinobacteriota, Chloroflexi, and Gemmatimonadota formed a relatively stable dominant bacterial assemblage, although the relative abundances of several phylum- and genus-level taxa varied among planting patterns. LEfSe analysis further identified potential biomarkers associated with different planting patterns, including *Conexibacter* in the pure *I. polycarpa* plantation, Saprospiraceae in the *I. polycarpa*–*Astragalus membranaceus* treatment, Kapabacteria-related taxa in the *I. polycarpa*–*Salvia rosmarinus* treatment, and Pedosphaeraceae-related taxa in the *I. polycarpa*–*Arachis hypogaea* treatment. FAPROTAX prediction indicated that chemoheterotrophic carbon utilization was the dominant functional potential of the soil bacterial communities, while nitrogen cycling-related functions also varied among understory planting patterns. Although the overall RDA models were not significant, single-factor fitting showed that SOM, pH, AN, and SWC were significantly associated with phylum-level bacterial community distribution, whereas AN was significantly associated with genus-level variation. Overall, appropriate understory plant configuration can improve soil conditions in young *I. polycarpa* plantations and may regulate bacterial community composition and functional potential. These findings provide a theoretical basis for understory plant selection and sustainable management of young *I. polycarpa* plantations in the Yellow River floodplain.

## Figures and Tables

**Table 1:** Soil physicochemical properties under different understory planting treatments in *I. polycarpa* plantations.

Index	IP	IP-AM	IP-IB	IP-SR	IP-AH
pH	7.48 ± 0.15 a	7.45 ± 0.03 a	7.44 ± 0.17 a	7.52 ± 0.12 a	7.61 ± 0.03 a
EC	0.77 ± 0.04 b	0.75 ± 0.01 b	0.74 ± 0.04 b	1.10 ± 0.09 a	0.79 ± 0.06 b
SWC	7.06 ± 0.61 c	10.1 ± 0.21 b	11.9 ± 0.69 a	7.49 ± 0.44 c	10.7 ± 0.19 b
SBD	1.16 ± 0.01 a	1.16 ± 0.01 a	1.16 ± 0.01 a	1.17 ± 0.01 a	1.18 ± 0.01 a
SOM	9.48 ± 0.32 c	12.3 ± 0.34 b	10.6 ± 0.38 c	14.3 ± 0.41 a	14.5 ± 0.30 a
AN	67.9 ± 0.68 c	80.0 ± 0.48 a	73.8 ± 0.36 b	74.5 ± 0.23 b	81.3 ± 0.37 a
AP	10.2 ± 0.10 c	11.9 ± 0.94 bc	13.6 ± 0.38 a	13.5 ± 0.23 a	12.7 ± 0.15 ab
AK	103 ± 5.77 b	90.0 ± 1.36 c	90.4 ± 1.20 c	123 ± 5.77 a	90.3 ± 3.54 c

Note: Values are presented as mean ± standard deviation, *n* = 3. Different lowercase letters within the same row indicate significant differences among treatments at *p* < 0.05. pH, soil acidity or alkalinity; EC, soil electrical conductivity; SWC, soil water content; SBD, soil bulk density; SOM, soil organic matter; AN, alkali-hydrolyzable nitrogen; AP, available phosphorus; AK, available potassium. IP, pure *I. polycarpa* plantation; IP-AM, *I. polycarpa* + *Astragalus membranaceus*; IP-IB, *I. polycarpa* + *Ipomoea batatas*; IP-SR, *I. polycarpa* + *Salvia rosmarinus*; IP-AH, *I. polycarpa* + *Arachis hypogaea*.

## Data Availability

The raw 16S rRNA gene amplicon sequencing data generated in this study have been deposited in the NCBI Sequence Read Archive (SRA) under BioProject accession number PRJNA1503785 and SRA study accession number SRP722399.

## Appendix A

**Table A1:** ASV numbers and taxonomic annotation statistics of soil bacterial communities under different understory planting treatments.

Treatment	Total ASVs	Unique ASVs	Domain	Phylum	Class	Order	Family	Genus	Species
IP	14,308	8836	252	138	286	1289	2189	8771	748
IP-AM	13,363	8038	199	141	281	1174	2021	8141	790
IP-IB	11,846	6473	159	147	211	1088	1601	7267	704
IP-SR	12,965	7864	227	120	250	1123	1934	8167	779
IP-AH	13,533	8708	361	239	333	1100	1926	8388	758
